# Oncological outcomes in an Australian cohort according to the new prostate cancer grading groupings

**DOI:** 10.1186/s12885-017-3533-9

**Published:** 2017-08-10

**Authors:** K. R. Beckmann, A. D. Vincent, M. E. O’Callaghan, P. Cohen, S. Chang, M. Borg, S. M. Evans, D. M. Roder, K. L. Moretti, for the South Australia Prostate Cancer Clinical Outcomes Collaborative

**Affiliations:** 1 Centre for Population Health Research, Sansom Institute for Health Research, University of South Australia, Adelaide, Australia; 2 South Australian Prostate Cancer Clinical Outcomes Collaborative, Repatriation General Hospital, Adelaide, Australia; 3 School of Medicine, University of Adelaide, Adelaide, Australia; 4 Flinders Centre for Innovation in Cancer, Flinders University, Adelaide, Australia; 5 SA Pathology, Health SA, Adelaide, Australia; 6 Adelaide Radiotherapy Centre, Adelaide, Australia; 7 Department of Epidemiology and Preventive Medicine, Monash University, Melbourne, Australia

**Keywords:** Prostate cancer, Grade groups, Clinical outcomes, Survival, Biochemical recurrence

## Abstract

**Background:**

A new 5-tiered grading grouping system has recently been endorsed for reporting of prostate cancer (PCa) grade to better reflect escalating risk of progression and cancer death. While several validations of the new grade groupings have been undertaken, most have involved centralised pathological review by specialist urological pathologists.

**Methods:**

Participants included 4268 men with non-metastatic PCa diagnosed between 2006 and 2013 from the multi-institutional South Australia Prostate Cancer Clinical Outcomes Collaborative registry. PCa-specific survival and biochemical recurrence-free survival were compared across the five grade groups using multivariable competing risk regression.

**Results:**

For the entire cohort, risk of PCa death increased with increasing grade groups (at biopsy) Adjusted subdistribution-hazard ratios [sHR] and 95% confidence intervals [95%CI] were: 2.2 (1.5–3.6); 2.5 (1.6–4.2); 4.1 (2.6–6.7) and 8.7 (4.5–14.0) for grade groups II (pattern 3 + 4), III (pattern 4 + 3), IV (total score 8) and V (total score 9–10) respectively, relative to grade group I (total score < =6). Clear gradients in risk of PCa death were observed for radical prostatectomy (RP), but were less clear for those who had radiotherapy (RT) with curative intent and those who were managed conservatively. Likewise, risk of biochemical recurrence increased across grade groups, with a strong and clear gradient for men undergoing RP [sHR (95%CI): 2.0 (1.4–2.8); 3.8 (2.9–5.9); 5.3 (3.5–8.0); 11.2 (6.5–19.2) for grade groups II, III, IV and V respectively, relative to grade group I], and a less clear gradient for men undergoing RT.

**Conclusion:**

In general, the new five-tiered grade groupings distinguished PCa survival and recurrence outcomes for men with PCa. The absence of a clear gradient for RT may be due to heterogeneity in this patient group.

## Background

Histological grade is an important prognostic indicator for prostate cancer (PCa) and is used extensively in defining risk categories for disease progression, along with other clinical characteristics, to guide treatment decisions and follow-up care [1–3]. The Gleason grading system developed 50 years ago, has been the universally adopted grading system for PCa, and has undergone a number of modifications. Major changes introduced in 2005 [4] led to significant upward shift in grade assignment from that time [5, 6].

Since then, a new more ‘patient friendly’ system for categorising prostate cancer grade, originally proposed by Epstein [7], has been endorsed by the International Society of Urological Pathologists (ISUP) [8]. The new grading system proposes reporting grade according to 5 risk groups reflecting an escalating risk of progression and cancer death, namely grade group I (Gleason ≤3 + 3 = 6), grade group II (Gleason 3 + 4 = 7); grade group III (Gleason 4 + 3 = 7); grade group IV (total Gleason score = 8); and grade group V (total Gleason Score = 9–10). Separating total Gleason score of 7 into patterns 3 + 4 and 4 + 3 provides official recognition of the prognostic differences between these designations [7, 9–12], differences which have long been recognised and considered by clinicians in determining treatment options. A further distinction has been made between total Gleason scores 8 and 9–10, which are generally grouped together as a single high risk category in most risk classification systems.

One of the key motivations for reclassifying grade into these five new groups is to better convey to a non-clinical audience the level of risk associated with disease grade. Labelling the lowest grade category as grade group I rather than Gleason Score of 6, provides a greater sense of lower risk of disease progression, and may help some men accept a recommendation for active surveillance rather than definitive treatment in the first instance.

Several validation studies have confirmed the predictive accuracy of the new grade groupings for biochemical recurrence (BCR) in international cohorts, both for men undergoing radical prostatectomy (RP) [7, 13–15] and radiotherapy treatment (RT) [13, 16, 17]. The new grade groupings have also been validated with respect to risk of prostate cancer death [18]. These include two recently published Australian validation studies which examined the performance of the new five-tier grade groupings in both men undergoing RP [15] and men undergoing RT in a trial setting [17]. All of these studies included centralised assessment or review of biopsy specimens. In the community setting multiple pathology services are engaged in assessing grade at biopsy and on RP specimens, and not all cases undergo specialist uro-pathological review. Consequently grade reported to clinicians and patients is not standardised and may not be uniform. Hence, it is also important to examine the applicability of the proposed new grading groupings in the context of non-centralised grading in a community based setting.

To this end, the aim of this study was to examine oncological outcomes, i.e. risk of PCa mortality and biochemical recurrence [BCR], according to new five-tier grade groupings for different management approaches, within a multi-institutional, community-based cohort from Australia.

## Methods

### Data source and subjects

The South Australian Prostate Cancer Clinical Outcomes Collaborative (SA-PCCOC) database is a long running prospective clinical registry which collects tumour characteristics, treatment details and oncological and functional outcome data for men with PCa across both the public and private sector in South Australia [19]. The study sample included all men in the SA-PCCOC registry with localised PCa diagnosis between 2006 and 2013 who had biopsy Gleason grade patterns recorded. During this period, registry coverage was approximately 50% of all cases in the state and included recruitment from all public hospitals, which are government run with universal access for all Australians, as well as approximately 50% of private urologists/urology services. Diagnoses before 2006 were excluded to limit cases to those graded after ISUPs revision of the grading system in 2005. Men with evidence of metastatic disease (clinical or imaging) at or within 45 days of diagnosis were also excluded, since metastatic disease may distort outcome assessment by grade.

### Measures

Data on patient characteristics including age at diagnosis, public or private health care management, place of residence; clinical features including grade, prostate specific antigen [PSA] levels, stage, and symptomatic presentation (i.e. referral due to symptoms - i.e. lower urinary tract symptoms, haematuria, bone pain – versus referral for elevated PSA), primary and subsequent treatment modalities, and dates of biochemical recurrence and death were extracted from SA-PCCOC for eligible cases. An area level measure of socioeconomic status was derived from patient’s residential postcode, using the Australian Bureau of Statistics Index of Socioeconomic Advantage and Disadvantage [20]. Death data were obtained from both the South Australian Register of Births, Deaths, and Marriages and the National Death Index.

For analyses of outcomes among men receiving curative treatment, we restricted the cohort to men who received curative RP or RT within 12 months of diagnosis. RT included external beam radiotherapy (EBRT), brachytherapy, or a combination of both. Conservative management was defined as management via watchful waiting (WW), active surveillance (AS) or androgen deprivation therapy (ADT) alone.

Grade at diagnosis, grouped according to the recently endorsed five-tiered system [8], was the key variable of interest in this study. For comparative purposes only biopsy grade was considered across all treatment groups including radical prostatectomy. Key outcomes in this study were prostate cancer-specific survival (PCSS) and biochemical recurrence-free survival (BRFS). PCSS was defined as the time from diagnosis to death, where PCa was indicated on the death certificate as a primary contributing cause of death. BRFS was defined as the time from date of diagnosis to first evidence of biochemical recurrence (BCR) among men who underwent definitive treatment. BCR was defined for patients receiving RP as two consecutive PSA values of >0.2 ng/mL [21], and for those receiving primary radiation therapy, any PSA increase >2 ng/mL higher than the post-RT PSA nadir value, regardless of the serum concentration of the nadir [22]. Survival durations were calculated from the date of diagnosis until the date of BCR, death or censoring date of June 30, 2016 (i.e. most recent deaths/PSA update), which ever was earliest.

### Analysis

Descriptive analyses of demographic, clinical and treatment characteristics according to grade groups were undertaken, with extended Wilcoxon rank-sum tests used to assess trends across ordered groups. Survival outcomes were initially assessed using Kaplan-Meier methods with log rank tests for differences in survival by grade groups. For Kaplan-Meier curves and log rank analyses competing risks are censored.

PCSS and BRFS were also compared across biopsy grade groupings (I to V) using univariable and multivariable competing risk regression, according to Fine and Gray’s methodology [23], with death from causes other than PCa as the competing risk. We undertook analyses for the entire cohort as well as for separate treatment subgroups: conservative management, RP and curative RT. All regression models controlled for age at diagnosis (continuous), year of diagnosis (continuous), public or private healthcare management, closest preceding PSA level to diagnosis (<10, 10- < 20, 20 + ng/ml), clinical stage (<cT3 v cT3+), symptomatic presentation (yes/no), and where appropriate, specific treatment types (e.g. robot-assisted versus open surgery, brachytherapy versus EBRT, ADT) and total dose received in Grays (continuous) for RT patients. Wald’s test was used to test for significant trends across grade groups in multivariable models. The potential for pairwise interactions between grade and other baseline factors was explored using likelihood ratio tests, comparing nested models with and without interactions. Statistically significant interactions were observed for treatment approaches and grade, in relation to both PCa mortality (*p* = 0.03), and BCR (*p* < 0.001). We therefore report results of subgroup analyses for different treatment modalities.

Due to known inaccuracies in assessing grade at biopsy, a sensitivity analyses was also undertaken for the subset who underwent RP, comparing the discriminatory power of prostatectomy versus biopsy grade to predict biochemical recurrence via the Akaike Information Criteria (AIC) in separate multivariable models.

Statistical analyses were undertaken using Stata v 12.1 [24].

## Results

### Clinical characteristics

Data were available for a total of 4268 men, diagnosed between 2006 and 2013. Two thirds of patients were classified as grade group I or II on biopsy, according to the new grade groupings. The mean age at diagnosis increased with increasing grade, as did median PSA at diagnosis (see Table 1). The proportion of men presenting with symptoms at diagnosis also increased with increasing grade groupings. However, grade groups did not differ with respect to number of cores taken at biopsy (median = 12). In the case of grade group V, a higher proportion was managed in the public system compared with other grade groups.

**Table 1:** Cohort characteristics by the new 5 tier Grade Groups (at biopsy)

	Grade Groups					
Characteristics (*N* = 4268)	I	II	III	IV	V	p-value^a^
Total - *n* (%)	1782 (42)	1154 (27)	647 (15)	399 (9)	286 (7)	
Clinical characteristics						
Mean age - *years (SD)*	66 (9)	67 (9)	70 (10)	72 (9)	74 (10)	<0.001
Median PSA - *ng/mL (IQR)*	7 (5–10)	8 (6–12)	10 (7–16)	11 (7–20)	17 (8–20)	<0.001
Public patient - *no. (%)*	952 (53)	594 (51)	316 (49)	223 (56)	190 (66)	<0.001
Presented with symptoms - *no. (%)*	395 (22)	229 (20)	112 (17)	80 (20)	87 (30)	<0.001
Primary Treatment - *n* (%)						
Radical Prostatectomy^b^	719 (40)	592 (51)	260 (40)	130 (33)	39 (14)	<0.001
Radiotherapy^b,c^ (with curative intent)	488 (27)	356 (31)	243 (38)	136 (34)	111 (38)	<0.001
Observation (AS or WW)	488 (27)	109 (9)	75 (12)	48 (12)	49 (17)	<0.001
ADT alone	31 (2)	54 (5)	47 (7)	62 (15)	71 (25)	<0.001
Outcomes						
PCa deaths^d^ – *no. (%)*	40 (2)	52 (5)	42 (7)	48 (12)	77 (27)	<0.001
Other deaths^d^ – *no. (%)*	157 (9)	115 (10)	75 (12)	61 (15)	34 (12)	<0.001
Biochemical recurrence^d^ – *no. (%)*	104 (11)	136 (17)	124 (29)	70 (30)	44 (37)	<0.001
PCa survival (5 yrs) - *% (95% CI)*	98 (97–99)	96 (95–97)	95 (93–96)	84 (80–88)	64 (59–69)	<0.001
BCR free survival (5 yrs)^e^ - *% (95% CI)*	91 (89–93)	84 (81–86)	73 (68–77)	70 (63–76)	67 (57–75)	<0.001

Corresponding Gleason patterns/scores for grade groups I-V are: 3 + 3, 3 + 4, 4 + 3, 8, 9–10

^a^ *P*-values from: log rank tests for survival & nonparametric tests for trend across ordered groups

^b^Includes RP/ curative RT at any time after diagnosis

^c^RT includes external beam, brachytherapy, or combination of both

^d^Total number of events during the follow-up period

^e^Biochemical recurrence among men who received definitive treatment (with PSA follow-up data) *n* = 2770

As expected, treatment patterns varied considerably across grade groups. RP was the primary treatment in 40% of men with grade group I and 51% with grade group II, but only 14% for men with grade group V disease. RT as the primary treatment was less variable. The proportion receiving RT with curative intent ranged from 27% for grade group I to 38% for grade group III and 39% for grade group V. Twenty seven percent of men in grade group I and 10% in grade group II underwent observation without immediate treatment.

Five-year PCSS decreased from 98% (95% CI 97–99%) for grade group I to 64% (95% CI 59–69%) for grade group V. Five-year BRFS decreased from 91% (95% CI 89–93) among men in grade group I to 67% (95% CI 57–75) for grade group V. The median follow-up time for the whole cohort was 72 months (inter-quartile range 52–96 months).

### Prostate cancer specific survival

**Fig. 1:**
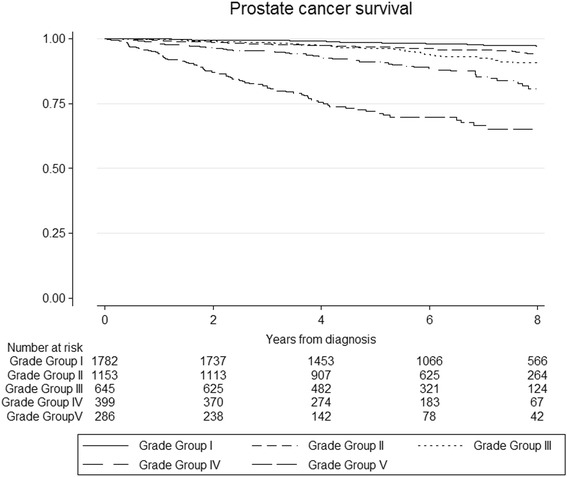
Unadjusted Kaplan-Meier survival curves for prostate cancer specific mortality, by grade groups at biopsy. [Grade I = (3 + 3); Grade II = (3 + 4); Grade III = (4 + 3); Grade IV = (total score = 8); Gleason 5 = (total score = 9–10)]

Figure 1 presents Kaplan-Meier PCa-specific survival curves for each of the new five-tier grade groupings I – V for the whole cohort. Survival decreased incrementally with higher grade grouping, as expected. Table 2 presents results of unadjusted and covariate adjusted competing risk regressions for PCa mortality across grade groups. Adjustment for covariates attenuated the effect of grade on risk of PCa death, in some instances quite considerably. This is likely to be explained by associations with other prognostic factors (age and PSA levels) across grade groups. Using backwards elimination modelling we confirmed that differences in age and pre-treatment PSA levels were the main factors contributing to the attenuation effect. Even so, grade remained the strongest independent predictor of death from PCa.

**Table 2:** Risk of prostate cancer death by the new 5-tier grade groups within treatment subgroups

Treatment sub-group	No. Events/Total	Competing risk regression			
		Unadjusted sHR (95%CI)	*p*-value	Adjusted sHR^a^ (95%CI)	*p*-value
All men	259/4264				
Grade group I (3 + 3)	40/1780	1.0	-	1.00	
Grade group II (3 + 4)	52/1154	2.1 (1.4–3.2)	<0.001	2.2 (1.5–3.6)	<0.001
Grade group III (4 + 3)	42/645	3.2 (2.1–4.9)	<0.001	2.5 (1.6–4.2)	<0.001
Grade group IV (8)	48/399	6.1 (4.0–9.2)	<0.001	4.1 (2.6–6.7)	<0.001
Grade group V (9–10)	77/286	16.8 (11.4–24.7)	<0.001	8.7 (5.4–14.0)	<0.001
*P for trend*			<0.001		<0.001
Conservative management^b^	136/1033				
Grade group I (3 + 3)	19/518	1.0	-	1.00	
Grade group II (3 + 4)	25/163	4.3 (2.4–7.8)	<0.001	3.0 (1.5–5.8)	0.001
Grade group III (4 + 3)	17/122	3.9 (2.1–7.4)	<0.001	2.2 (1.0–4.8)	0.04
Grade group IV (8)	26/110	6.9 (3.8–12.4)	<0.001	4.1 (2.0–8.4)	<0.001
Grade group V (9–10)	49/120	15.2 (8.9–25.8)	<0.001	8.5 (4.2–17.2)	<0.001
*P for trend*			<0.001		<0.001
Radical prostatectomy	22/1624				
Grade group I (3 + 3)	4/637	1.0	-	1.0	-
Grade group II (3 + 4)	5/568	1.7 (0.5–6.2)	0.44	1.6 (0.4–6.1)	0.48
Grade group III (4 + 3)	3/254	2.4 (0.5–10.5)	0.26	2.3 (0.5–10.9)	0.29
Grade group IV (8)	6/126	9.5 (2.6–33.8)	0.001	9.5 (2.6–35.8)	0.001
Grade group V (9–10)	4/39	29.1 (7.1–120)	<0.001	28.4 (6.4–124)	<0.001
*P for trend*			<0.001		<0.001
Curative radiotherapy	75/1143				
Grade group I (3 + 3)	15/385	1.0	-	1.00	-
Grade group II (3 + 4)	19/314	1.8 (0.9–3.5)	0.10	1.4 (0.7–3.0)	0.37
Grade group III (4 + 3)	19/218	2.7 (1.4–5.5)	0.003	2.3 (1.1–4.8)	0.03
Grade group IV (8)	10/125	2.6 (1.2–5.8)	0.02	2.0 (0.8–7.8)	0.13
Grade group V (9–10)	12/101	4.2 (2.0–9.0)	<0.001	2.8 (1.2–6.8)	0.02
*P for trend*			<0.001		<0.001

^a^sHR: Sub-distribution hazard ratios derived from competing risk regression adjusted for age, year of diagnosis, diagnostic PSA, clinical stage, area level SES, public /private management, treatment modality (appropriate to subgroups)

^b^The conservative management group consists of men who were managed through watchful waiting, active surveillance or androgen deprivation therapy alone

Within the entire cohort, risk of PCa death increased incrementally with increasing grade group, independently of other factors, based on multivariable competing risk regression (adjusted sub-distribution hazard ratios [sHR] = 2.2 (1.5–3.6); 2.5 (1.6–4.2); 4.1 (2.6–6.7) and 8.7 (4.5–14.0) for grade groups II, III, IV and V respectively, relative to grade group I. A similar gradient in risk of PC death was observed among men who - who underwent RP. For men managed conservatively and men who received curative RT, a clear increase in SHRs across grade groups was not evident, however *p*-values for trend were statistically significant in all treatment subgroups.

### Biochemical recurrence-free survival

**Fig. 2:**
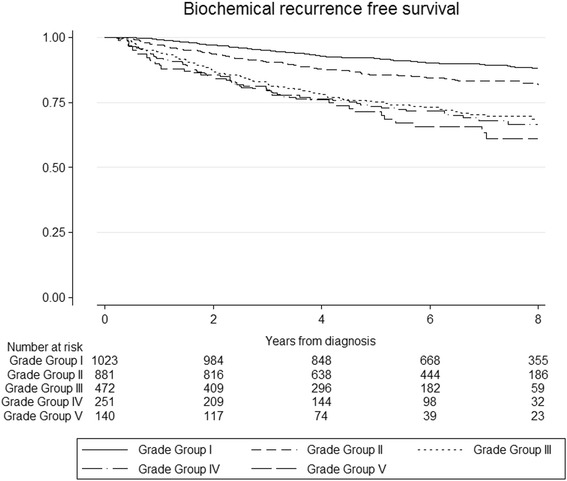
Unadjusted Kaplan-Meier survival curves for biochemical recurrence-free survival, by grade groups at biopsy. [Grade I = (3 + 3); Grade I I = (3 + 4); Grade III = (4 + 3); Grade IV = (total score = 8); Gleason 5 = (total score = 9–10)]

Figure 2 presents survival curves for BCR by grade groups for patients treated curatively, indicating poorer outcomes with increasing grade group. Results from competing risk regression analyses for BCR by grade groups are shown in Table 3. These analyses show a clear gradient in risk of BCR across grades following RP (p for trend < 0.001). For patients who received curative RT, we did not observe a constant increase in sHRs across grade groups, though the trend overall was statistically significant (<0.001). Including type of RT (EBRT versus brachytherapy), total dose and concurrent or adjuvant ADT in the model did not alter this pattern. Likewise, findings did not change when men receiving neo-adjuvant or adjuvant ADT were excluded.

**Table 3:** Risk of biochemical recurrence (BCR) by the new five-tier grade groupings among men having definitive treatment

Treatment subgroups	No. Events/Total	Competing risk regression			
		Unadjusted sHR (95%CI)	*p*-value	Adjusted sHR^a^ (95%CI)	*p*-value
Radical prostatectomy	292/1351				
*Clinical grade groups N* = 1351					
Grade group I (3 + 3)	57/541	1.0	-	1.0	-
Grade group II (3 + 4)	94/468	2.0 (1.5–2.9)	<0.001	2.0 (1.4–2.8)	<0.001
Grade group III (4 + 3)	73/200	4.1 (2.9–5.9)	<0.001	3.8 (2.9–5.9)	<0.001
Grade group IV (8)	47/108	5.5 (3.7–8.0)	<0.001	5.3 (3.5–8.0)	<0.001
Grade group V (9–10)	21/34	9.5 (5.6–16.1)	<0.001	11.2 (6.5–19.2)	<0.001
*P for trend*			<0.001		<0.001
Curative radiotherapy *N* = 937	185/937				
Grade group I (3 + 3)	46/321	1.0	-	1.0	-
Grade group II (3 + 4)	42/254	1.2 (0.8–19)	0.291	1.2 (0.7–1.8)	0.32
Grade group III (4 + 3)	51/187	2.4 (1.8–3.6)	<0.001	2.5 (1.5–3.7)	<0.001
Grade group IV (8)	23/97	2.0 (1.2–3.4)	0.006	2.0 (0.9–2.9)	0.02
Grade group V (9–10)	23/78	2.4 (1.8–5.2)	<0.001	2.4 (1.4–4.1)	0.001
*P for trend*			<0.001		<0.001

Cases were excluded if <2 post- treatment PSA measures were recorded (273 (17%) cases excluded for prostatectomy group; 206 (18%) cases excluded for radiotherapy subgroup)

^a^sHR: Sub-distribution hazard ratios derived from competing risk regression adjusted for age, year of diagnosis, diagnostic PSA, clinical stage, area level SES, and public/private management

### Incremental comparison between grade groups

To specifically compare outcomes between incremental grade groups we repeated multivariable competing risk regression models with each grade group referenced to the previous grade grouping (Table 4). With respect to PCa death we observed non-significant trends toward higher risk for Grade group III compared with II among men undergoing radical prostatectomy (sHR = 1.4, CI 0.3-6.5) and men receiving curative radiotherapy (sHR = 1.6, CI 0.9–3.1). The lack of statistical significance is likely to be due to the low number of PCa deaths among men in these grade groupings. With respect to BCR, comparisons showed statistically significant higher risk of progression for grade group III compared with grade group II for both treatment groups (RP: sHR = 2.1, CI 1.5–2.8; RT: sHR = 2.0, CI 1.3–3.1). Comparisons of grade groups IV and grade group III indicated statistically significant differences for risk of PCa death for men undergoing RP (sHR = 4.2, CI 1.0–17.1) and men managed conservatively (sHR = 1.9, CI 1.0–3.4) but not for men undergoing curative RT, and no difference in relation to risk of BCR for either curative approach. Risk of PCa death and BCR were both elevated for men in grade group V compared with IV for RP and RT patients, but the difference only reached statistical significance in relation to BCR for men undergoing RP. Among men who were managed conservatively we observed a significant difference in risk of PCa death for grade group V compared grade group IV (sHR = 2.1, CI 1.3–3.4), but saw no difference in risk death between grade groups II and III).

**Table 4:** Risk of prostate cancer (PCa) death and biochemical recurrence (BCR), relative to previous grade grouping, by biopsy grade

Treatment sub-group	PCa deaths		BCR^a^	
	Adjusted sHR (95%CI)	*p*	Adjusted sHR^b^ (95%CI)	*p*
Radical prostatectomy				
Grade group I (3 + 3)	-		-	-
Grade group II (3 + 4)	1.6 (0.4–6.1)	0.48	2.0 (1.4–2.8)	<0.001
Grade group III (4 + 3)	1.4 (0.3–6.5)	0.64	2.1 (1.5–2.8)	<0.001
Grade group IV (8)	4.2 (1.0–17.1)	0.05	1.4 (0.9–2.0)	0.12
Grade group V (9–10)	3.0 (0.8–10.9)	0.10	1.8 (1.0–3.1)	0.05
Curative radiotherapy				
Grade group I (3 + 3)	-		-	
Grade group II (3 + 4)	1.4 (0.7–3.0)	0.37	1.2 (0.7–1.8)	0.32
Grade group III (4 + 3)	1.6 (0.9–3.1)	0.13	2.0 (1.3–3.1)	0.001
Grade group IV (8)	0.9 (0.4–2.0)	0.74	0.8 (0.5–1.4)	0.41
Grade group V (9–10)	1.4 (0.6–3.5)	0.46	1.3 (0.7–2.4)	0.46
Conservative management^c^				
Grade group I (3 + 3)	-			
Grade group II (3 + 4)	3.0 (1.5–5.8)	0.001	Not applicable	
Grade group III (4 + 3)	0.7 (0.4–1.4)	0.35	-	
Grade group IV (8)	1.9 (1.0–3.4)	0.04	-	
Grade group V (9–10)	2.1 (1.3–3.4)	0.004	-	

^a^For analysis of BCR, cases were excluded if <2 post- treatment PSA measures were recorded (273 (17%) cases excluded for prostatectomy group; 206 (18%) cases excluded for radiotherapy subgroup)

^b^sHR: Subdistribution-hazard ratios derived from multivariable competing risk regression adjusted for age, year of diagnosis, diagnostic PSA, clinical stage, area level SES, and public/private management

^c^The conservative management group consists of men who were managed through watchful waiting, active surveillance or androgen deprivation therapy alone

### Sensitivity analyses

Comparison of biopsy and prostatectomy grade groups among men who underwent radical prostatectomy indicated that prostatectomy grade was marginally superior to biopsy grade in predicting BCR following RP, as indicated by the difference in AIC for the two models (Table 5).

**Table 5:** Comparison of biopsy grade and prostatectomy grade in predicting risk of progression among men who underwent radical prostatectomy

Grade groups (*n* = 1334)	Biopsy grade grouping			Prostatectomy grade grouping		
	No. events/total	Adjusted sHR^a^ (95% CI)	*p*-value	No. events/total	Adjusted sHR^a^ (95% CI)	*p*-value
	292/1334			292/1334		
Grade group I (3 + 3)	57/532	1.0	−	19/286	1.0	−
Grade group II (3 + 4)	94/462	2.0 (1.5–2.9)	<0.001	87/595	2.4 (1.5–3.9)	0.001
Grade group III (4 + 3)	73/199	4.3 (3.0–6.2)	<0.001	119/321	7.6 (4.6–12.4)	<0.001
Grade group IV (8)	47/108	5.8 (3.9–8.7)	<0.001	25/62	9.0 (4.8–17.0)	<0.001
Grade group V (9–10)	1/34	10.4 (5.9–18.2)	<0.001	42/70	16.8 (9.6–29.7)	<0.001
P for trend			<0.001			<0.001
AIC^b^			3934			3882
Change in AIC			+52			0

Models only include cases with detail on both biopsy and prostatectomy grade sufficient to determine ISUP-2014 groups and ≥2 post-treatment PSA measures

^a^sHR: subdistribution Hazard ration from multivariable competing risk regression models adjusted for age, pre-treatment PSA, clinical evidence of extra prostatic disease, symptomatic presentation, public or privately managed

^b^Lower (AIC: Akaike’s Information Criterion) indicates better discriminatory power for prostatectomy grade compared with biopsy grade groups

## Discussion

The new 5-tiered grade groups (determined at biopsy) correlated well with increasing risk of PCa mortality and risk of disease progression in most instances. Although adjustment for other prognostic factors attenuated differences across grade groups, grade was a strong predictor of disease specific outcomes in our cohort. In general, these findings indicate the generalizability of findings from validation studies with standardised pathology undertaken by specialist urological pathologists [7, 13, 17, 25] to community practice with non-centralised pathology undertaken predominantly by non-specialist pathologists. Even so, there is room for improvement in diagnostic methods, given that grade assessed on radical prostatectomy specimens better discriminated of the risk of BCR than grade assessed at biopsy.

Our study confirms the widely reported findings by others [7, 10–12] which indicate that grade groups II and III (which previously were often grouped together as a total Gleason score = 7) confer different levels of risk of BCR among men undergoing RP or RT. Our results also support making a distinction between a total Gleason score of 8 (grade group IV) and scores of 9 and 10 (grade group V) [26, 27], since risk of BCR is higher for grade group V compared with IV for both treatment subgroups. Among men managed conservatively, statistically significant differences were observed between grade groups IV and V, but not between grade groups II and III. The lack of distinct difference may be due to the very mixed nature of the cohort being managed conservatively. An examination of the differences in outcomes between patterns (3 + 5 vs 4 + 4 vs 5 + 3) within grade group IV, which remains controversial [28, 29], was beyond the scope of this paper.

A clear gradient of worsening outcomes was observed with increasing grade group among men receiving RP, but was less prominent for men undergoing curative RT. Risk of BCR among men undergoing curative RT was effectively identical for grade III to V, contrary to expectations. This irregularity was not explained by differences in RT dose or treatment type. Results remained similar when models included receipt of concurrent/adjuvant ADT and also when those who received adjuvant therapy were excluded. Interestingly, others reporting outcomes across grade groups among men undergoing RT have also not shown a clear gradient, similar to our results [13, 16, 17]. Possible reasons for the lack of a clear gradient in outcomes among RT patients include: 1) the influence of other unmeasured confounders, given the mixed characteristics within the subgroup receiving RT, e.g. those with higher risk disease as well as those with lower risk disease who were not fit for surgery; 2) incorrect assignment of grade at biopsy with potentially higher levels of misclassification, since RT patients tended to be older and have higher PSA levels which are both associated with upgrading [30]), or 3) different effects of RT for different grade groups, that is, RT may be more effective for higher and less effective for lower grade tumours leading to less distinct survival curves.

Among men managed conservatively, there was a clear difference in risk of PCa mortality between grade groups I and II. This result provides some support for AS among men whose tumour are classified as grade group I, provided other prognostic indicators are favourable. Conversely, a case could be made for actively treating men with grade II disease if they are fit for surgery, since prostate cancer survival among those who were managed conservatively was significantly worse for grade group II compared with I. Currently, some guidelines recommend offering AS for favourable intermediate risk (grade group II) disease, determined largely by the extent of Gleason pattern 4 [31, 32]. Our data do not offer this level of granularity. Furthermore, interpretation of outcomes for the conservatively managed group is difficult given this subgroup of patients includes a mix of lower risk cases under active surveillance and older higher risk patients undergoing watchful waiting and/or intermittent hormone treatment. (Data on intent of conservative management approaches were not available for the entire study period, hence further subdivision was not possible). This mix of patient characteristics may be contributing to or masking differences across grade groups.

Finally further research is needed to develop new risk stratification tools for disease progression/PCa mortality based on new grade classifications in combination with other clinical characteristics, e.g. diagnostic PSA levels, to provide patients and clinicians with more refined risk-based information to guide treatment decisions.

### Limitations

In undertaking this study, we did not commission a review of the original grade assignment but rather reclassified groupings based on recorded primary and secondary Gleason patterns. Nor did we include tertiary pattern 5 in grade classification, as it was not always recorded.

Also, we were unable to account for potential confounding by factors such as comorbidity and frailty, due to a lack of information on these measures. Comorbidity/frailty may be contributing to poorer clinical outcomes for men in the lower grade groups, particularly those undergoing radiotherapy or conservative management due to being unfit for surgery. Not being able to account for these factors may have obscured the influence of grade, leading to less clear incremental effect across grade groups in these treatment subgroups. This is supported by the smaller effect sizes for grade among men receiving RT compared to those observe for the RP subgroup.

Since state-wide coverage was only 50% and private patients were likely underrepresented in SA-PCCOC during the study period, our results may be affected by selection bias. Given public patients are likely to be older and have more comorbidities and, or more advanced disease, the likely impact of such bias would be toward reduced strength of association between grade groups and clinical outcomes. This may be another factor explaining the less clear distinction in men receiving RT or managed conservatively.

The strengths of this study in relation to assessing applicability of the new grading classification in a community setting are a relatively long follow-up time and multi-institutional nature of our cohort.

## Conclusion

The newly proposed five-tier grade groupings distinguish risk of disease progression and PCa mortality reasonably accurately in our cohort, with the exception of some anomalies in relation to disease progression following RT and for men managed conservatively. This may be due to heterogeneity with respect to other factors within these groups. In general our findings indicate the applicability of the new grade group, assigned in the context of non-standardised assessment of grade across multiple practices in a community based setting.

These results lend support to the adoption of the new grading classification, whereby men with low risk (grade group I) disease may be encouraged to consider surveillance in the first instance.

## References

[1] National Comprehensive Cancer Network (2015). NCCN clinical Proactice guidelines in oncology: prostate cancer. In*.*, vol. v1.2015.

[2] D'Amico AV, Whittington R, Malkowicz SB, Schultz D, Blank K, Broderick GA, Tomaszewski JE, Renshaw AA, Kaplan I, Beard CJ (1998). Biochemical outcome after radical prostatectomy, external beam radiation therapy, or interstitial radiation therapy for clinically localized prostate cancer. JAMA.

[3] Cooperberg MR, Pasta DJ, Elkin EP, Litwin MS, Latini DM, Du Chane J, Carroll PR (2005). The University of California, san Francisco cancer of the prostate risk assessment score: a straightforward and reliable preoperative predictor of disease recurrence after radical prostatectomy. J Urol.

[4] Epstein JI, Allsbrook WC, Amin MB, Egevad LL, Committee IG (2005). The 2005 International Society of Urological Pathology (ISUP) consensus conference on Gleason grading of prostatic carcinoma. Am J Surg Path.

[5] Billis A, Guimaraes MS, Freitas LL, Meirelles L, Magna LA, Ferreira U (2008). The impact of the 2005 international society of urological pathology consensus conference on standard Gleason grading of prostatic carcinoma in needle biopsies. J Urol.

[6] Danneman D, Drevin L, Robinson D, Stattin P, Egevad L (2015). Gleason inflation 1998-2011: a registry study of 97,168 men. BJUI.

[7] Pierorazio PM, Walsh PC, Partin AW, Epstein JI (2013). Prognostic Gleason grade grouping: data based on the modified Gleason scoring system. BJUI.

[8] Epstein JI, Egevad L, Amin MB, Delahunt B, Srigley JR, Humphrey PA (2016). The 2014 International Society of Urological Pathology (ISUP) consensus conference on Gleason grading of prostatic carcinoma: definition of grading patterns and proposal for a new grading system. Am J Surg Path.

[9] Alenda O, Ploussard G, Mouracade P, Xylinas E, de la Taille A, Allory Y, Vordos D, Hoznek A, Abbou CC, Salomon L (2011). Impact of the primary Gleason pattern on biochemical recurrence-free survival after radical prostatectomy: a single-center cohort of 1,248 patients with Gleason 7 tumors. World J Urol.

[10] Helpap B, Ringli D, Shaikhibrahim Z, Wernert N, Kristiansen G (2013). The heterogeneous Gleason 7 carcinoma of the prostate: analyses of low and high grade (risk) carcinomas with criteria of the International Society of Urological Pathology (ISUP). Path Res Pract.

[11] Rusthoven CG, Waxweiler TV, DeWitt PE, Flaig TW, Raben D, Kavanagh BD (2015). Gleason stratifications prognostic for survival in men receiving definitive external beam radiation therapy for localized prostate cancer. Urol Oncol.

[12] Stark JR, Perner S, Stampfer MJ, Sinnott JA, Finn S, Eisenstein AS, Ma J, Fiorentino M, Kurth T, Loda M (2009). Gleason score and lethal prostate cancer: does 3 + 4 = 4 + 3?. J Clin Oncol.

[13] Epstein JI, Zelefsky MJ, Sjoberg DD, Nelson JB, Egevad L, Magi-Galluzzi C, Vickers AJ, Parwani AV, Reuter VE, Fine SW (2016). A contemporary prostate cancer grading system: a validated alternative to the Gleason score. Eur Urol.

[14] Spratt DE, Cole AI, Palapattu GS, Weizer AZ, Jackson WC, Montgomery JS, Dess RT, Zhao SG, Lee JY, Wu A (2016). Independent surgical validation of the new prostate cancer grade-grouping system. BJUI.

[15] Samaratunga H, Delahunt B, Gianduzzo T, Coughlin G, Duffy D, LeFevre I, Johannsen S, Egevad L, Yaxley J (2015). The prognostic significance of the 2014 International Society of Urological Pathology (ISUP) grading system for prostate cancer. Pathology.

[16] Spratt DE, Jackson WC, Abugharib A, Tomlins SA, Dess RT, Soni PD, Lee JY, Zhao SG, Cole AI, Zumsteg ZS (2016). Independent validation of the prognostic capacity of the ISUP prostate cancer grade grouping system for radiation treated patients with long-term follow-up. Prostate Cancer Prostatic Dis.

[17] Delahunt B, Egevad L, Srigley JR, Steigler A, Murray JD, Atkinson C, Matthews J, Duchesne G, Spry NA, Christie D (2015). Validation of International Society of Urological Pathology (ISUP) grading for prostatic adenocarcinoma in thin core biopsies using TROG 03.04 'RADAR' trial clinical data. Pathology.

[18] Berney DM, Beltran L, Fisher G, North BV, Greenberg D, Moller H, Soosay G, Scardino P, Cuzick J (2016). Validation of a contemporary prostate cancer grading system using prostate cancer death as outcome. Brit J Cancer.

[19] Beckmann K, Pinnock C, Tamblyn DJ, Kopsaftis T, Stapleton AM, Roder DM (2009). Clinical and socio-demographic profle of an Australian multi-institutional prostate cancer cohort. Asia-Pacific J Clin Oncol.

[20] Australian Bureau of Statistics (2008). Information Paper: An Introduction to Socio-Economic Indexes for Areas (SEIFA) 2006. In Canberra.

[21] Stephenson AJ, Kattan MW, Eastham JA, Dotan ZA, Bianco FJ, Lilja H, Scardino PT (2006). Defining biochemical recurrence of prostate cancer after radical prostatectomy: a proposal for a standardized definition. J Clin Oncol.

[22] Roach M, Hanks G, Thames H, Schellhammer P, Shipley WU, Sokol GH, Sandler H (2006). Defining biochemical failure following radiotherapy with or without hormonal therapy in men with clinically localized prostate cancer: recommendations of the RTOG-ASTRO phoenix consensus conference. Int J Rad Oncol Biol Phys.

[23] Fine JP, Gray RJ (1999). A proportional hazards model for the subdistribution of a competing risk. J Am Stat Ass.

[24] StataCorp (2010). Stata Statisitical Software: Release 12.

[25] Epstein JI (2016). New prostate cancer grade group system correlates with prostate cancer death in addition to biochemical recurrence. Brit J Cancer.

[26] Lim SK, Kim KH, Shin TY, Chung BH, Hong SJ, Choi YD, Rha KH (2013). Gleason 5+4 has worse oncological and pathological outcomes compared with Gleason 4+5: significance of Gleason 5 pattern. Ann Surg Oncol.

[27] Nanda A, Chen MH, Renshaw AA, D'Amico AV (2009). Gleason pattern 5 prostate cancer: further stratification of patients with high-risk disease and implications for future randomized trials. Int J Rad Oncol Biol Phys.

[28] van den Bergh RC, van der Kwast TH, de Jong J, Zargar H, Ryan AJ, Costello AJ, Murphy DG, van der Poel HG (2016). Validation of the novel International Society of Urological Pathology 2014 five-tier Gleason grade grouping: biochemical recurrence rates for 3+5 disease may be overestimated. BJUI.

[29] Srigley JR, Delahunt B, Egevad L, Samaratunga H, Yaxley J, Evans A (2016). One is the new six: the International Society of Urological Pathology (ISUP) patient-focused approach to Gleason grading. Can Urol Ass J.

[30] Epstein JI, Feng Z, Trock BJ, Pierorazio PM (2012). Upgrading and downgrading of prostate cancer from biopsy to radical prostatectomy: incidence and predictive factors using the modified Gleason grading system and factoring in tertiary grades. Eur Urol.

[31] Chen RC, Rumble RB, Loblaw DA, Finelli A, Ehdaie B, Cooperberg MR, Morgan SC, Tyldesley S, Haluschak JJ, Tan W (2016). Active surveillance for the Management of Localized Prostate Cancer (Cancer Care Ontario guideline): American Society of Clinical Oncology clinical practice guideline endorsement. J Clin Oncol.

[32] National Collaborating Centre for Cancer (2014). Prostate cancer: diagnosis and treatment. In: Report No: Clinical guideline; no 175.

